# Leveraging Technology for Vestibular Assessment and Rehabilitation in the Operational Environment: A Scoping Review

**DOI:** 10.3390/bioengineering11020117

**Published:** 2024-01-25

**Authors:** Carrie W. Hoppes, Karen H. Lambert, Susan L. Whitney, Isaac D. Erbele, Carlos R. Esquivel, Tony T. Yuan

**Affiliations:** 1 Army-Baylor University Doctoral Program in Physical Therapy, 3630 Stanley Road, Joint Base San Antonio-Fort Sam Houston, TX 78234, USA; 2 Hearing Center of Excellence, 2200 Bergquist Drive, Lackland Air Force Base, TX 78236, USA; karenlambert@zcorebusiness.com; 3 Department of Physical Therapy, School of Health and Rehabilitation Sciences, University of Pittsburgh, Bridgeside Point 1, 100 Technology Drive, Pittsburgh, PA 15219, USA; whitney@pitt.edu; 4 Department of Otolaryngology-Head and Neck Surgery, San Antonio Uniformed Services Health Education Consortium, Brooke Army Medical Center, 3551 Roger Brooke Drive, Joint Base San Antonio-Fort Sam Houston, TX 78234, USA; isaac.d.erbele.mil@health.mil; 5 Department of Surgery, School of Medicine, Uniformed Services University of the Health Sciences, 4301 Jones Bridge Road, Bethesda, MD 20814, USA; 6 Wilford Hall Ambulatory Surgical Center, 2200 Bergquist Drive, Lackland Air Force Base, TX 78236, USA; carlos.r.esquivel.civ@health.mil; 7 Department of Radiology and Radiological Sciences, School of Medicine, Uniformed Services University of the Health Sciences, 4301 Jones Bridge Road, Bethesda, MD 20814, USA; tony.yuan@usuhs.edu

**Keywords:** vestibular, assessment, rehabilitation, operational medicine, austere medicine

## Abstract

Introduction: The vestibular system, essential for gaze and postural stability, can be damaged by threats on the battlefield. Technology can aid in vestibular assessment and rehabilitation; however, not all devices are conducive to the delivery of healthcare in an austere setting. This scoping review aimed to examine the literature for technologies that can be utilized for vestibular assessment and rehabilitation in operational environments. Materials and Methods: A comprehensive search of PubMed was performed. Articles were included if they related to central or peripheral vestibular disorders, addressed assessment or rehabilitation, leveraged technology, and were written in English. Articles were excluded if they discussed health conditions other than vestibular disorders, focused on devices or techniques not conducive to the operational environment, or were written in a language other than English. Results: Our search strategy yielded 32 articles: 8 articles met our inclusion and exclusion criteria whereas the other 24 articles were rejected. Discussion: There is untapped potential for leveraging technology for vestibular assessment and rehabilitation in the operational environment. Few studies were found in the peer-reviewed literature that described the application of technology to improve the identification of central and/or peripheral vestibular system impairments; triage of acutely injured patients; diagnosis; delivery and monitoring of rehabilitation; and determination of readiness for return to duty. Conclusions: This scoping review highlighted technology for vestibular assessment and rehabilitation feasible for use in an austere setting. Such technology may be leveraged for prevention; monitoring exposure to mechanisms of injury; vestibular-ocular motor evaluation; assessment, treatment, and monitoring of rehabilitation progress; and return-to-duty determination after vestibular injury. Future Directions: The future of vestibular assessment and rehabilitation may be shaped by austere manufacturing and 3D printing; artificial intelligence; drug delivery in combination with vestibular implantation; organ-on-chip and organoids; cell and gene therapy; and bioprinting.

## 1. Introduction

The vestibular system, comprised of the semicircular canals, otolith organs, and eighth cranial nerves in the peripheral system, and the brainstem, brain, and cerebellum in the central system, is essential for gaze and postural stability. It allows service members to keep their eyes fixed on a target while their head is moving, and additionally contributes to the maintenance of balance. The vestibular system is especially important for balance under conditions where vision is obscured (e.g., smoke, darkness, use of night vision goggles) or the support surface is challenging or moving (e.g., walking over rocks and debris, standing in the turret of a military vehicle). This system can be damaged by threats on the battlefield, from blast waves [1] to directed energy technologies [2,3].

Akin et al. [4] reported that 5–57% of individuals with dizziness post-concussion are diagnosed with benign paroxysmal positional vertigo (BPPV). Weakness on caloric testing (a marker of horizontal semicircular and/or superior vestibular nerve dysfunction) occurred in 3–51% of individuals with dizziness post-concussion [4]. Ocular motor abnormalities (a marker of central nervous system dysfunction) occurred in ≤8% of individuals with dizziness post-concussion [4], though one study included in the review reported a frequency of 45% [5]. Dizziness and imbalance can occur as a result of blast exposure [6,7]. In individuals with dizziness and imbalance following blast exposure, the frequency of BPPV was 3–20% and the frequency of weakness on caloric testing was 0–40% [4]. The frequency of ocular motor abnormalities was 3–4% [4], though one study included in the review reported a frequency of 45% [8]. Dizziness and unsteadiness have also been reported by individuals exposed to a sound/pressure phenomenon in Havana, Cuba in 2016–2017 [2], colloquially referred to as “Havana Syndrome”. Regardless of the cause, physical and occupational therapists are well suited to evaluate and treat individuals with damage to the vestibular system who have body structure and function impairments, activity limitations, and/or participation restrictions [9].

Evaluation of the vestibular system requires a systematic assessment of the visual, vestibular, and balance systems. Technology can aid in the assessment of nystagmus (resulting from an imbalance of vestibular nuclei firing or abnormal stimulation of one or more semicircular canals). Video Frenzel goggles prevent visual fixation (which can mask nystagmus originating from the peripheral system) to allow the clinician to better visualize and quantify eye movements. Force plates, inertial measurement units (IMUs), and motion capture equipment can be used to measure kinematics of balance and gait. Similarly, vestibular rehabilitation can be delivered via smartphone- or tablet-based devices. Virtual reality systems and head-mounted devices can also be used to deliver or augment traditional vestibular rehabilitation [10]. Early vestibular rehabilitation may result in better outcomes [11].

While technology can assist physical and occupational therapists in performing vestibular assessment and rehabilitation, not all such technologies are conducive to delivery of healthcare in an operational environment. In this context, the environment is characterized by the presence of extreme conditions and constrained resource availability. Electrical power and internet may be unreliable or absent, the climate may be variable (with extremes in temperature), and clinical settings may lack level floors for the use of force plates. Dirt, dust, and moisture can have negative effects on electronic devices. Medical care may be constrained by limited time, large numbers of casualties, and security concerns. Medical assets may need to be rapidly maneuverable to remain close to service members engaged in combat, precluding use of large, heavy, and non-portable equipment. Access to specialty providers may be limited or non-existent. This scoping review aimed to examine the literature for technologies that can be utilized for vestibular assessment and rehabilitation in operational environments. Understanding the technology gap of existing vestibular assessment and rehabilitation technologies is critical for innovation and development in the field, aiming to significantly improve warfighter readiness and return-to-duty on the battlefield.

## 2. Materials and Methods

To achieve this aim, a comprehensive search of PubMed was performed from the earliest dates through October 2023. A Population, Intervention, Comparison and Outcomes (PICO) framework was used to develop a search strategy: for patients with central or peripheral vestibular disorders (P), what technologies can be utilized for vestibular assessment and rehabilitation in operational environments (I), in comparison to standard care (C), to improve warfighter readiness and return-to-duty on the battlefield (O)? The following search strategy was used: “vestibular” AND (assess* OR rehab*) AND (“military medicine” OR “operational medicine” OR “austere medicine” OR “disaster medicine”).

Articles were included if they related to central (disorders of the brainstem, brain, and cerebellum; e.g., mild traumatic brain injury or concussion, vestibular migraine, etc.) or peripheral (disorders of the eighth cranial nerves and distal structures [12]; e.g., BPPV, vestibular neuronitis, labyrinthitis, labyrinthine concussion, perilymphatic fistula, superior semicircular canal dehiscence syndrome, etc.) vestibular disorders, addressed assessment or rehabilitation, leveraged technology, and were written in English. Articles were excluded if they discussed health conditions other than vestibular disorders, focused on devices or techniques not conducive to the operational environment, or were written in a language other than English. Titles and/or abstracts of studies retrieved using the search strategy were screened to identify studies that potentially met the inclusion criteria outlined earlier. The full text of these potentially eligible studies was then retrieved and assessed for eligibility. Charting data from the included articles was performed independently; obtaining and confirming data from the authors/investigators was not performed. Data sought included the population and technology used. Assumptions were made by the authors on the potential application of the technology in the operational environment. These data were summarized in a table and organized alphabetically by the lead author’s surname. The Preferred Reporting Items for Systematic reviews and Meta-Analyses Extension for Scoping Reviews (PRISMA-ScR) checklist [13] was used in the development of this scoping review.

## 3. Results

**Figure 1:**
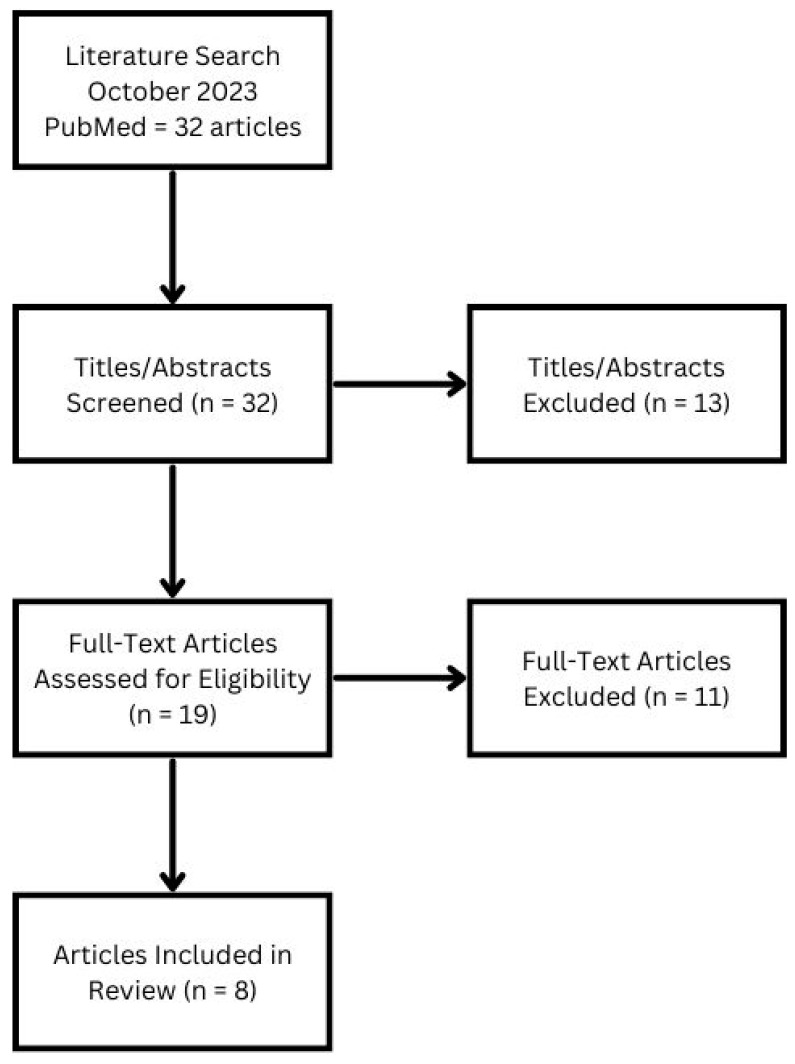
Flowchart of the literature search sequence.

Our search strategy yielded 32 articles: 8 articles [14,15,16,17,18,19,20,21] met our inclusion and exclusion criteria whereas the other 24 articles were rejected (Figure 1). Reasons for rejection included: discussed health conditions other than central or peripheral vestibular disorders (2), failed to leverage technology (5), focused on devices or techniques not conducive to an operational environment (10), described symptoms or populations (6), and were written in a language other than English (1). We also hand searched the reference list of the included studies identified through the search. The 8 articles that related to the application of technology for vestibular assessment and rehabilitation and were feasible for use in an operational environment are highlighted in Table 1.

Ten articles that focused on devices or techniques not conducive to an operational environment, based on expert opinion and the collective personal experience of the authors in managing individuals with vestibular disorders, were rejected from this scoping review. One study [22] leveraged magnetic resonance spectroscopic imaging that is not currently available or conducive for use in operational environments. Similarly, three studies [23,24,25] leveraged the Computer Assisted Rehabilitation Environment (CAREN) system (Motek Medical, Houten, The Netherlands). The size and cost of the CAREN system, in addition to the need for a specialized engineer operator to build scenarios and run the system, make it ill-suited for use in operational environments. Two studies [26,27] used a rotary chair in a light-proof enclosure for vestibular function testing; such diagnostic testing equipment could not be readily installed in an austere setting. Finally, four articles [28,29,30,31] described elaborate, inter-disciplinary specialty rehabilitation programs delivered stateside. The Comprehensive Combat and Complex Casualty Care (C5) center located at Naval Medical Center San Diego, CA, describes a model of care using 18 medical providers from 10 different specialties [28]. The National Intrepid Center of Excellence (NICoE) [29] located at Walter Reed National Military Medical Center, MD, and its satellite Warrior Recovery Center (WRC) [30] located at Fort Carson, CO, similarly offer an inter-disciplinary approach to assessment and rehabilitation. Resource-intensive models such as these would not be feasible in operational settings where access to specialty providers may be limited or non-existent. The Military Functional Assessment Program (MFAP) is a five-day return-to-duty assessment that includes items such as the Ropes Confidence Course (obstacle course) [31], making it unfeasible in an operational environment.

## 4. Discussion

Only a few studies were found in the peer-reviewed literature that describe the application of technology to improve the prevention of vestibular injury; identification of central and/or peripheral vestibular system impairments; triage of acutely injured patients; diagnosis; delivery and monitoring of rehabilitation; and determination of readiness for return to duty. Given the limited literature on the topic, there are a number of unmet gaps in understanding the untapped potential for leveraging technology for vestibular assessment and rehabilitation in the operational environment. There are also gaps in transitioning currently validated technologies and developing new technologies for use in austere settings.

### 4.1. Prevention

The prevention of vestibular injuries might be an overlooked topic, given the limited literature and available technologies, but could serve as a critical unmet need for military medicine. Although there is no conclusive evidence that helmets can prevent vestibular injury, it would stand to reason that they may afford some protection to the skull (the vestibular system is located in the petrous portion of the temporal bone of the skull) and brain. A systematic review on the effects of noise exposure on the vestibular system reported an association between noise over-exposure and vestibular dysfunction [32]. There is no conclusive evidence that hearing protection can prevent vestibular injury, but given the proximity of the cochlea and labyrinth, perhaps it may afford some protection. Hearing protection should be worn during all live-fire events to include combat [33]. However, wear of hearing protection while on patrol can be as low as 4% [34]. Jones and Pearson reported a decline in wear of hearing protection in at least one ear from 39% to 12% following a health promotion activity [35]. Reasons for non-use can include decreased situational awareness (interference with detecting and localizing auditory warnings), impaired communication (exchanging information and hearing verbal orders), and poor fit (devices were incompatible with other gear and/or difficult to fit) [36]. In addition to military personal protective equipment, there are several promising therapeutic approaches that might also afford some prevention of vestibular injury. Jiang et al. [37] conducted a review on protection of vestibular hair cells. Promising strategies for preventing loss or damage of vestibular hair cells include pharmacotherapy (such as induction of heat shock proteins, antioxidant treatments, use of antiapoptotic agents, use of insulin-like growth factor-1, use of other protective agents or molecules, or a combination of different drugs) or gene therapy [37].

### 4.2. Monitoring Exposure

Vartanian et al. [18] leveraged blast gauges (BlackBox Biometrics, LLC, Rochester, NY, USA) to record changes in overpressure and acceleration during breacher training exercises. Advanced technological devices have the potential to yield valuable data regarding blast exposures, while simultaneously serving as an early warning system to prompt medical providers to conduct injury screening when specific pre-defined thresholds are reached. For instance, when the green status light-emitting diodes change to yellow or red, this can be a visual indicator to the service member, unit personnel and leaders, and medical personnel that the service member should be removed from duty and screened for vestibular impairments post-blast. Blast exposure data could be correlated with qualitative and quantitative measurements of vestibular system function. Such devices may be leveraged for monitoring exposure to repetitive, low-level blasts as well. External sensors data from the Blast Gauge^®^ System do not necessarily align with physiological symptoms or clinical outcomes. While Suttles [17] commented on the measurement of head impact severity using telemetry, these devices have low specificity in predicting concussion [38]. However, as technology continues to advance and direct relationships are drawn between exposure parameters and physiological responses, perhaps blast gauges and helmet-based sensors can mature to have greater clinical utility in the future.

### 4.3. Vestibular-Ocular Motor Evaluation

A thorough examination of the vestibular system should include the evaluation of eye movements (such as in response to positional testing) with fixation removed. Halmagyi et al. have deemed them “essential for any clinician dealing with dizzy patients” [39]. While our literature search did not find any articles leveraging video Frenzel goggles (video nystagmography [VNG]) in the operational environment, there are numerous companies that have developed goggles that could be used in an austere setting. Low-technology, low-cost options that can be carried in a uniform pocket or medical kit are also available [40,41,42].

Similarly, the video Head Impulse Test (vHIT) is becoming the standard of care for identifying an acute unilateral vestibulopathy [43]. Several companies have developed vHIT goggles, which enable the visualization of overt and covert saccades, as well as calculation of vestibular-ocular reflex (VOR) gain. Some vHIT goggles may also offer the Suppression Head Impulse (SHIMP) test [44], which may eliminate covert saccades for more precise measurement of VOR gain [45]. Parker et al. [46] developed a custom app that used an iPhone Xs (Apple, Inc., Cupertino, CA, USA) to perform the vHIT. While using a smartphone is novel, the collected data had to be manually postprocessed and analyzed. Depending on operational security restrictions, smartphone-based assessment of the VOR may be possible in the operational environment, but automated data analysis and easy-to-interpret visualization of results would be critical. Kuroda et al. [47] developed a prototype iPhone-vHIT system that is inexpensive and portable, and may hold promise for testing in austere settings.

Both VNG and vHIT technologies are valuable in the operational environment to improve assessment, especially to determine the origin (whether the condition is peripheral or central) and urgency of the underlying condition. In an operational environment, the basic assessment can consist of a Head Impulse Nystagmus Test of Skew (HINTS) exam [48], which can include horizontal canal vHIT, and positional (Dix-Hallpike and Roll) tests for BPPV. The VNG and vHIT devices would need to be ruggedized to withstand extremes in temperature, as well as dirt, dust, and moisture. Battery back-up would be beneficial in an austere setting where electrical power may be unreliable or absent. Other technologies like caloric, ocular vestibular-evoked myogenic potential (VEMP), and rotary chair testing are not practical in austere environments.

While cervical VEMP, auditory brainstem response (ABR), and electrocochleography (ECoG) are not technologies for vestibular-ocular motor evaluation, they are often employed in hospital and research settings as part of an assessment battery. However, they are not practical in austere environments. Quantitative electroencephalogram (EEG), especially now that it can be portable, may have promise in aiding diagnosis of central vestibular disorders (e.g., concussion). Brown et al. [49] conducted a review of emerging techniques in sport-related concussion; they noted that use “depends on well-trained personnel to ensure quality data acquisition, and hands-on review to ensure that artifacts are identified and removed”. As EEG technology and algorithms continue to advance, perhaps it will have greater clinical utility in the future.

### 4.4. Assessment, Treatment and Monitoring Rehabilitation Progress

Gera et al. [14] and Martini et al. [15] leveraged IMUs (Opal; APDM Wearable Technologies, Inc., Portland, OR, USA) to instrument the modified Clinical Test of Sensory Integration and Balance. Inertial measurement units may reveal subtle qualities of movement that are imperceptible in the absence of such instrumentation, thereby enhancing the assessment and monitoring of rehabilitation progress. Several companies offer ruggedized IMUs that could be used for precision measurements in an operational environment. Ruggedized IMUs are designed to withstand harsh environments and extreme conditions, such as high shock and vibration, extremes in temperature, and exposure to dirt, dust, and moisture.

Patterson et al. [50] conducted a pilot study using the SWAY Balance Mobile Application (SWAY Medical, Tulsa, OK, USA) on an Apple iPod Touch (Apple Computer Inc., Cupertino, CA, USA). Dewan et al. [51] later explored use of the SWAY Balance™ Mobile Application (version 2.1.1, SWAY Medical, Tulsa, OK, USA) on a smartphone held with both hands against the chest to provide objective measurement of thoracic sway during a series of balance tasks. EQ Balance (Highmark Interactive Inc., Oakville, ON, Canada) also uses a smartphone held with both hands against the chest to provide objective measurement of performance during a series of balance tasks and offers remote monitoring of patient performance. These types of assessment and monitoring systems leverage the accelerometers, gyroscopes, and/or magnetometers contained in smartphones and tablets. Depending on operational security restrictions, smartphone-based assessment of postural sway may be possible in the operational environment. Their use for telemedicine may allow for remote assessment and delivery of balance rehabilitation. This may be especially helpful in an operational environment where access to specialty providers (e.g., physical therapists) may be limited or non-existent.

The Barany Society Classification OverSight Committee has not established specific diagnostic criteria for cervical dizziness [52]. Despite this, it is commonplace to evaluate cervical range of motion and joint position sense during the assessment of individuals with cervical dizziness. Bagaianu et al. [21] leveraged the Zebris CMS 20 (Zebris Medizinetechnik GmbH, Isny, Germany), configured using a helmet and a thoracic belt that were each fitted with three ultrasound microphones to determine three-dimensional head-on-body motion, to evaluate joint position sense. This device relies on the timing of the intervals between the emission and the reception of ultrasound pulses to measure distances to the microphones [21]. It can accurately and reliably measure cervical spine range of motion [53]. However, it is not known how the battlefield environment with its high intensity and/or impulse noise or other ambient noise might influence device usability. With regard to measurement of range of motion and joint position sense, a device such as the NeckCare™ System (NeckCare, Minneapolis, MN, USA) could allow for assessment of range of motion and joint position sense in an operational environment. Advanced technological devices, like the ones noted, can enhance the precision of clinical measurements for assessment and monitoring of rehabilitation progress, which could be critical in providing data for return-to-duty decisions.

Whitney et al. [20] developed VestAid (BlueHalo, Rockville, MD, USA), a tablet device that utilizes eye and facial recognition software to record head velocities and eye-gaze accuracy while patients perform gaze stability exercises. Their small case series demonstrates that advanced technological devices can enhance the precision of clinical measurements for assessment and monitoring of rehabilitation progress. A device such as the Bertec Vision Advantage (Bertec Corporation, Columbus, OH, USA) could allow for an assessment of the VOR using the Dynamic Visual Acuity and Gaze Stabilization Test paradigms in an operational environment.

Depending on operational security restrictions, smartphone-based assessment and rehabilitation may be possible in the operational environment. Noda et al. [54] conducted a scoping review on devices and apps for taking a patient history and recording subjective symptoms, objective testing, diagnosis, and treatment of vestibular dysfunction. Such devices and apps may be used for telemedicine, allowing for remote assessment and delivery of rehabilitation. This may be especially helpful in an operational environment where access to specialty providers (e.g., audiologists, otolaryngologists, and neurologists) may be limited or non-existent. Shah et al. [55] found that eye movements recorded using a smartphone camera during the Dix-Hallpike test could be remotely assessed by neuro-otologists. Young et al. [56] used custom-made lightweight swimming goggles with monocular infrared lights attached to an audio/video recorder (Dizzy-cam video goggles) for vision-blocked assessment of vertigo attacks. Even when receiving care in-person, Kıroğlu and Dağkıran found that patients who were able to record their eye movements using a smartphone camera were diagnosed with Meniere’s disease sooner than those in a control group who did not record their eye movements (two attacks with a mean of 40 days in the video group and four attacks with a mean of 102 days in the control group, *p* < 0.001) [57].

Smartphone apps have also been developed for subjective visual vertical (SVV) testing (as an assessment of utricular function). Brodsky et al. found that a cutoff of >2.13° using the Visual Vertical (Clear Health Media, Wonga Park, Australia) app on an iPhone 5 (Apple, Cupertino, CA, USA) resulted in 66.7% sensitivity, 97.0% specificity, 80.0% positive predictive value, and 94.1% negative predictive value for detecting peripheral vestibular loss in pediatric patients [58]. Ulozienė et al. described the VIRVEST wearable virtual reality-based system for assessing SVV [59]. It consisted of a head-mounted display, Myo gesture control armband (Thalmic Labs Inc., Kitchener, ON, Canada) or general purpose gamepad (Red Samurai gamepad, GameStop Corp. Inc., Brampton, ON, Canada), and smartphone or tablet [59]. Similarly, Zabaneh et al. used a head-mounted display (C-SVV^®^ goggles) and OtoAccess™ software to assess SVV in patients with Meniere’s disease [60]. Zaleski-King et al. used a head-mounted display to assess SVV and the related Rod and Disk Test [61]. Given that the maculae of the otolith organs may be more vulnerable to pressure waves than the cristae of the semicircular canals [62], SVV may provide a quick injury screening when medical care is constrained by limited time or large numbers of casualties.

Meldrum et al. [63] developed a head-worn sensor and smartphone app for the delivery of vestibular rehabilitation (and they now also offer an associated clinician portal). The sensor (VG02; www.vertigenius.com accessed on 22 January 2024) uses an inertial measurement unit to measure the angular velocity of the head during gaze stabilization exercises [63]. A Bluetooth connection between the sensor and app allows the patient to receive real-time feedback on their exercise performance [63]. The Vertigenius device can enhance the precision of clinical measurements for assessment and monitoring of rehabilitation progress.

Nehrujee et al. [64] developed the VEstibular GAming System (VEGAS), consisting of a smartphone-based 3D virtual reality headset (Convergence VR Tech Labs Pvt. Ltd.) and two games for vestibular assessment and training. Serious games may encourage participation and adherence to the prescribed rehabilitation regimen; and game-based rewards for correct execution of gaze stability exercises may increase patient motivation and bolster performance. There are several smartphone applications that can be used with Google Cardboard (https://arvr.google.com/cardboard/ accessed on 22 January 2024) that could be utilized in austere environments, including VR Tunnel Race: Speed Rush VR (DTA Mobile, Cau Giay, Hanoi, Vietnam), VR XRacer: Racing VR Games (DTA Mobile, Cau Giay, Hanoi, Vietnam), VR RollerCoasters (VR Games Ltd.), and VR Escape Game (Blacksmith DoubleCircle).

### 4.5. Rehabilitation and Return-to-Duty Determination

Smith et al. [19] supported the use of virtual reality for the implementation of cognitive, visual, and vestibular training for returning service members to shooting and to duty. Virtual reality technology can be used to deliver rehabilitation in immersive and realistic environments where therapeutic exercises can be controlled, and warrior-specific tasks can be safely practiced. The virtual reality environment can also be manipulated to provide visual habituation for visually induced dizziness [65].

Prim et al. [16] mounted a near focus scope (10 × 40 Monocular; Barska, Pomona, CA, USA) onto a simulated weapon (Bluegun; Rings Manufacturing, Inc., Melbourne, FL, USA) combined with a computer display for the Run-Roll-Aim Task. They used technology for identification of impairments post-mild traumatic brain injury during a military-specific task. This performance-based task demonstrates leveraging technology to aid in return-to-duty determination.

## 5. Limitations

The major limitation of this scoping review is the lack of current evidence on the subject; only 8 articles were included in this scoping review. Perhaps other articles might have been identified if other databases or the gray literature was searched. Since none of the included studies specifically addressed our aim or PICO question, a critical appraisal of the articles was not performed. Instead, the included studies were used to shape our discussion of technologies that can be utilized for vestibular assessment and rehabilitation in operational environments. It was of greater importance to include potential technologies for future applications in austere settings, than to appraise the quality of how the technologies were applied in past, hospital- or research-based settings. Determination that the technology for vestibular assessment and rehabilitation was feasible for use in an operational environment was based on expert opinion and the collective personal experience of the authors in managing individuals with vestibular disorders. As data extraction was performed independently, there is also an increased risk of bias.

## 6. Conclusions

While many technologies can assist physical and occupational therapists in performing vestibular assessment and rehabilitation, not all such devices are conducive to delivery of healthcare in an operational environment. This scoping review highlighted technology for vestibular assessment and rehabilitation feasible for use in an austere setting. There is untapped potential for leveraging such technology for prevention; vestibular-ocular motor evaluation; monitoring exposure to mechanisms of injury; assessment, treatment, and monitoring of rehabilitation progress; and return-to-duty determination after vestibular injury.

## 7. Future Directions

Understanding the technology gap of existing vestibular assessment and rehabilitation technologies is critical for innovation and development in the field, aiming to significantly improve warfighter readiness and return-to-duty on the battlefield. The future of vestibular assessment and rehabilitation may leverage austere manufacturing and 3D printing; artificial intelligence; drug delivery in combination with vestibular implantation; organ-on-chip and organoids; cell and gene therapy; and bioprinting. While the use of all the following technologies may not be suitable for use in an austere setting, they will likely influence military medicine and the care of military personnel with vestibular disorders.

In the future, austere manufacturing and 3D printing may allow for the production of devices for vestibular assessment and rehabilitation (like Frenzel goggles). Not only are advances in hardware expected, but advances in software, too. Improved eye tracking and recording, with improved signal processing to remove motion artifacts, may allow for greater precision in VOR measurements. Artificial intelligence algorithms may be able to arrive at a diagnosis and rehabilitation strategy from features extracted from the patient’s history and response to questionnaires in combination with oculomotor, vestibulo-ocular, and postural stability measurements made by future devices. A web-based tool was developed by Dr. Devin McCaslin and colleagues at Mayo Clinic, Rochester, MN. It used a validated algorithm to extract features from reported symptoms and matched patients to the most appropriate clinical specialty. The tool was commercialized under the name DizzyGuide (https://dizzyguide.net/ accessed on 22 January 2024).

Intracochlear controlled release of medication has been demonstrated in combination with cochlear implantation [66]. Is intralabyrinthine medication delivery also possible in combination with vestibular implantation? Systematic reviews have found that vestibular implants can restore VOR function [67] and balance [68] in patients with bilateral vestibular loss. In the future, technological advances may enable an externally applied device to similarly replace lost vestibular function to expedite an injured service member’s return to duty. Presently, galvanic vestibular stimulation has been found to improve the results of vestibular rehabilitation [69,70]. It may be a promising modality to decrease imbalance post-injury.

Organ-on-chip and organoid biotechnology may also shape vestibular assessment and rehabilitation in the future. Mattei et al. [71] have derived inner ear organoids from human pluripotent stem cells. Such organoids may provide a means to study the effects of various injury mechanisms and disease processes, diagnostic imaging, and testing technologies, as well as preventative and restorative interventions.

Quan et al. [72] identified a combination of small molecules and small interfering ribonucleic acids capable of reprogramming adult cochlea hair cell-like cellular regeneration in mice with hair cell loss in vivo. In the future, can we similarly regenerate vestibular hair cells in animal models and eventually humans? Cell and gene therapy may augment or replace the need for vestibular rehabilitation. Bioprinting a replacement labyrinth may allow an otolaryngologist to restore vestibular system function to a service member injured on the battlefield in the far future.

## Figures and Tables

**Table 1:** Summary of articles that related to the application of technology for vestibular assessment and rehabilitation and were feasible for use in an operational environment.

Title and Authors	Population	Technology Used	Potential Application in the Operational Environment
Cervical Joint Position Sense in Hypobaric Conditions: A Randomized Double-Blind Controlled Trial [21]; Diana Bagaianu et al.	Healthy males (*n* = 36) 27.29 ± 9.71 years old recruited from the faculty, staff, students, and family of the students from a university and military hospital	Zebris CMS 20 (Zebris Medizinetechnik GmbH, Isny, Germany)	Measurement of range of motion and joint position sense for cervical dizziness
Inertial Sensor-Based Assessment of Central Sensory Integration for Balance After Mild Traumatic Brain Injury [14]; Geetanjali Gera et al.	Collegiate athletes who had sustained a mTBI within the past 2–3 days (*n* = 38; 20.6 ± 1.3 years old; 25 M/13 F) or who had not sustained a mTBI within the past 6 months (controls; *n* = 81; 21.0 ± 1.4 years old; 44 M/37 F)	Inertial measurement units (Opal; APDM, Inc., Portland, OR, USA)	Instrumented balance/gait measures for assessment and monitoring of rehabilitation progress
Symptoms and Central Sensory Integration in People With Chronic mTBI: Clinical Implications [15]; Douglas N. Martini et al.	41 people with chronic mTBI (39.8 ± 11.5 years old; 12M/29F) and 53 age- and sex-matched healthy controls (36.5 ± 12.1 years old; 21M/32F)	Inertial measurement units (Opals Version 1; APDM, Inc., Portland, OR, USA)	Instrumented balance/gait measures for assessment and monitoring of rehabilitation progress
Clinical Utility and Analysis of the Run-Roll-Aim Task: Informing Return-to-Duty Readiness Decisions in Active-Duty Service Members [16]; Julianna H. Prim et al.	33 people with mTBI (26.2 ± 5.2 years old; 31M/2F) and 50 healthy controls (30.2 ± 6.1 years old; 40M/10F)	Near focus scope (BARSKA Blueline 10 × 40 Monocular; Barska, Pomona, CA, USA) mounted on a simulated weapon (Bluegun; Rings Manufacturing, Inc., Melbourne, FL, USA) and computer display	Identification of impairments post-mTBI during a military-specific task to inform return-to-duty determination
Post-concussion Return to Shooting Progression for Military Service Members: A Scoping Review and Conceptual Framework [19]; Erin Smith et al.	Military service members (Scoping Review)	Virtual reality systems (in general)	Implementation of cognitive, visual, and vestibular training for returning service members to shooting and to duty
Potential of Visual Sensory Screening, Diagnostic Evaluation, and Training for Treatment of Postconcussive Symptoms and Performance Enhancement for Special Forces Qualified Personnel [17]; Sean T. Suttles	Special Operations Forces soldiers (Narrative Review)	Head Impact Telemetry System (Simbex, Lebanon, NH, USA); Nike Visual Sensory Training Stations and Nike SPARQ Package (Nike, Inc., Beaverton, OR, USA); Peripheral Awareness Trainer (Wayne Enterprises); Wayne Saccadic Fixator, Visual Choice Reaction Time Apparatus, and Multi-Domain Apparatus for Reaction Time (Lafayette Instrument Co., Lafayette, IN, USA); SVT (Sports Vision, Sydney, Australia); and Dynavision 2000 and D2 light boards (Dynavision International, LLC, Cincinnati, OH, USA)	Measurement of head impact features to quantify exposure and prompt injury screening; Identification and rehabilitation of visual impairments post-mTBI
Neuropsychological, Neurocognitive, Vestibular, and Neuroimaging Correlates of Exposure to Repetitive Low-Level Blast Waves: Evidence From Four Nonoverlapping Samples of Canadian Breachers [18]; Oshin Vartanian et al.	Male breachers (*n* = 70) and male Canadian Special Operations Forces Command members of similar average age with no experience in breaching (*n* = 14)	Blast gauges (BlackBox Biometrics, Rochester, NY, USA)	Measurement of changes in overpressure and acceleration to quantify exposure and prompt injury screening
Utility of VestAid to Detect Eye-Gaze Accuracy in a Participant Exposed to Directed Energy [20]; Susan L. Whitney et al.	Control (46 year old M), person exposed to directed energy (47 year old M), person post-concussion (19 year old F), and person with vestibular neuritis (71 year old F)	VestAid (Intelligent Automation dba BlueHalo, Rockville, MD, USA)	Instrumented gaze stability measures for assessment and monitoring of rehabilitation progress

F = female; M = male; mTBI = mild traumatic brain injury.
